# In Vivo Imaging Evaluation of Fluorescence Intensity at Tail Emission of Near-Infrared-I (NIR-I) Fluorophores in a Porcine Model

**DOI:** 10.3390/life12081123

**Published:** 2022-07-27

**Authors:** María Rita Rodríguez-Luna, Nariaki Okamoto, Mahdi Al-Taher, Deborah S. Keller, Lorenzo Cinelli, Anila Hoskere Ashoka, Andrey S. Klymchenko, Jacques Marescaux, Michele Diana

**Affiliations:** 1 Research Institute against Digestive Cancer (IRCAD), 1 Place de l’Hôpital, 67000 Strasbourg, France; nariaki.okamoto@ircad.fr (N.O.); mahdi.al-taher@ircad.fr (M.A.-T.); jacques.marescaux@ircad.fr (J.M.); michele.diana@ircad.fr (M.D.); 2 ICube Laboratory, Photonics Instrumentation for Health, 67081 Strasbourg, France; 3 Maastricht University Medical Center, 6229 Maastricht, The Netherlands; 4 Marks Colorectal Surgical Associates, Lankenau Medical Center, Main Line Health, Wynnewood, PA 19096, USA; debbykeller@gmail.com; 5 Department of Gastrointestinal Surgery, San Raffaele Hospital IRCCS, 20132 Milan, Italy; lorenzo.cinelli@ircad.fr; 6 Laboratoire de Bioimagerie et Pathologies, UMR 7021 CNRS, Université de Strasbourg, 74 Route du Rhin, 67401 Illkirch, France; anil.hoskere@gmail.com (A.H.A.); andrey.klymchenko@unistra.fr (A.S.K.)

**Keywords:** near-infrared window I (NIR-I), near-infrared window II (NIR-II), fluorescence imaging system, bile duct imaging, coated medical materials, indocyanine green, ICG, fluorescence image-guided surgery, FIGS

## Abstract

Over the last decade fluorescence-guided surgery has been primarily focused on the NIR-I window. However, the NIR-I window has constraints, such as limited penetration and scattering. Consequently, exploring the performance of NIR-I dyes at longer wavelengths (i.e., the NIR-II window) is crucial to expanding its application. Two fluorophores were used in three pigs to identify the mean fluorescence intensity (MFI) using two commercially available NIR-I and NIR-II cameras. The near-infrared coating of equipment (NICE) was used to identify endoluminal surgical catheters and indocyanine green (ICG) for common bile duct (CBD) characterization. The NIR-II window evaluation showed an MFI of 0.4 arbitrary units (a.u.) ± 0.106 a.u. in small bowel NICE-coated catheters and an MFI of 0.09 a.u. ± 0.039 a.u. in gastric ones. In CBD characterization, the ICG MFI was 0.12 a.u. ± 0.027 a.u., 0.18 a.u. ± 0.100 a.u., and 0.22 a.u. ± 0.041 a.u. at 5, 35, and 65 min, respectively. This in vivo imaging evaluation of NIR-I dyes confirms its application in the NIR-II domain. To the best of our knowledge, this is the first study assessing the MIF of NICE in the NIR-II window using a commercially available system. Further comparative trials are necessary to determine the superiority of NIR-II imaging systems.

## 1. Introduction

**Figure 1:**
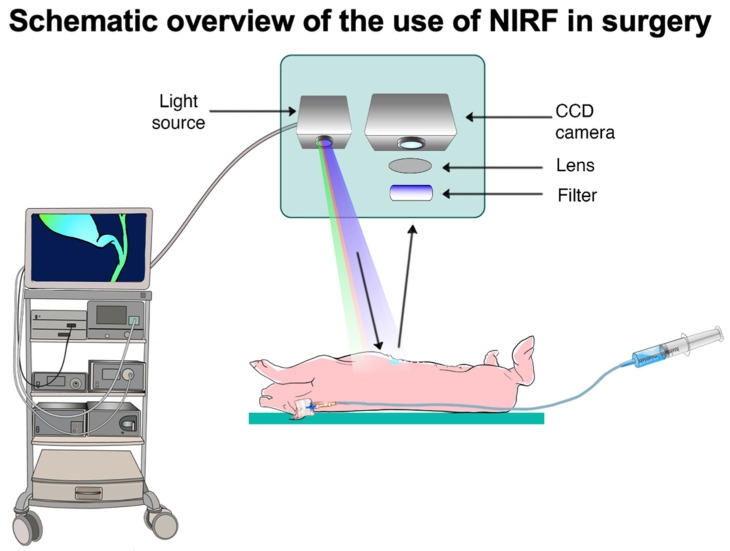
Schematic overview of the use of NIRF in surgery, classical view when using NIR-I imaging system.

Fluorescence image-guided surgery (FIGS) is a continuously evolving field. An increasing number of advanced technological imaging systems are being introduced for open, laparoscopic, and robotic surgery with the goal of increasing operative precision [1,2,3]. One tool used in this area is near-infrared (NIR) fluorescence imaging (FI) technology. NIR-FI allows for a real-time video rate visualization with a higher contrast and deeper penetration as compared to the visible light spectrum, allowing surgeons to better discern anatomical structures. NIR-FI requires the administration of a fluorescent contrast agent [4]. Essentially, in vivo NIR bioimaging can be understood in the context of light propagation. As described by Frangioni et al., a photon travels through tissue to reach the fluorescent contrast agent, and various results may occur depending on tissue scatter, anisotropy, and reflectance index. The same happens to the photon emitted by fluorophore. To excite and detect the spectral signal, the use of an optical imaging system is required [5] (Figure 1).

Over the past decade, FIGS has been mostly dedicated to what is known as the NIR-I window, which corresponds to a wavelength of 700 to 900 nm in the electromagnetic spectrum [4,6]. However, its performance in living tissues has some limitations, such as light absorption and loss of photons due to scattering. Indeed, these drawbacks increase exponentially with depth, allowing for tissue penetration of less than 1 cm, which restricts the NIR-I window capacity from detecting deeper anatomical features [7].

The first developed fluorophore, which gained Food and Drug Administration (FDA) approval for clinical use, was indocyanine green (ICG) [8]. ICG is a NIR-I dye, which corresponds to a peak fluorescent emission wavelength in the λ^ex^ max = 800 nm, λ^em^ max = 820 nm window. Since it binds tightly to plasma proteins, its half-life is 18 min, which is one of the main reasons for its extensive adoption as a navigational tool to assess superficial perfusion [9]. Due to its mostly hepatic clearance, ICG is also used to evaluate liver function and to characterize biliary anatomy. Lymphatic drainage mapping during oncological resections and tumoral margin recognition has also been described in the repertoire of applications [7].

ICG has not only been the most clinically used fluorophore [10] but it has also served as a cornerstone in the engineering of NIR imaging systems, especially those evaluating the NIR-I window. Recently, the ICG fluorescent emission spectra in the NIR-II window also referred as the “tail emission” or “off-peak NIR-I fluorescence emission” have been described [11]. In an in vivo small animal study, Starosolski et al. confirmed ICG’s applicability in quantitative bioimaging assessment in the NIR II-window using a custom-made InGaAS camera [12].

The goal of this preclinical large animal study is to evaluate the “tail emission” of two NIR-I fluorophores, namely ICG and near-infrared coating of equipment (NICE), using a quantitative assessment with mean fluorescence intensity (MFI) in the NIR-II window. For this purpose, ICG was intravenously (IV) administered as a contrast agent to characterize the common bile duct (CBD) and NICE was used as a catheter coating agent.

## 2. Materials and Methods

The study was conducted in strict accordance with the recommendations published in the Guide for the Care and Use of Laboratory Animals of the National Institutes of Health. The present study is part of the ELIOS protocol (Endoscopic Luminescent Imaging for Oncology Surgery), fully approved by the local Ethical Committee on Animal Experimentation (ICOMETH No. 38.2016.01.085), and by the French Ministry of Superior Education and Research (MESR) (APAFIS#8721-2017013010316298-v2). All sections of this report adhere to the ARRIVE Guidelines for reporting animal research [13].

This experimental study included three pigs (Sus scrofa domesticus, ssp. Large White, both genders, mean weight: 32 ± 4.54 kg). The 3Rs principle (replacement, refinement, and reduction) were followed in compliance with the optimal animal welfare conditions [14].

Animals fasted for 24 h before surgery. They received an intramuscular (IM) injection of Zolazepam + Tiletamine 10 mg/kg (Zoletil ND, Virbac, France) as premedication. Anesthesia was induced by means of an IV injection of Propofol 3 mg/kg (Propofol Lipuro ND, B Braun, France) + Rocuronium 0.8 mg/kg (Esmeron ND, MSD, France), hence allowing for intubation and mechanical ventilation. Pigs were sedated during the experiment via an inhalation of isoflurane 2–3% (Isoflurin ND, Axience France) + Oxygen. Buprenorphine (Buprecare ND, Axience, France) at 0.01 mg/kg, administered intramuscularly, was used as a painkiller. At the end of the experiments, the pigs were euthanized under deep anesthesia (isoflurane 5%) with a lethal IV injection of Pentobarbital 40 mg/kg (Exagon ND, Axience, France).

### 2.1. ICG Dye Preparation

ICG (Infracyanine^®^, Serb laboratories, Paris, France) was prepared according to the manufacturer’s specifications, diluting the powder in 5% glucose solution to a final dilution of 25 mg/10 mL. The ICG dose was calculated according to the animal’s weight (0.10 mg/kg).

### 2.2. Fluorescent NICE-Coated Catheter Preparation

The near-infrared coating of equipment (NICE) was synthesized by combining a biocompatible polymer poly (methyl methacrylate) (PMMA) with a specifically engineered fluorescent dye [15]. NICE is an ultrabright and stable biocompatible fluorescent coating that can coat surgical devices of any material and be visualized using commercially available surgical NIR cameras. NICE displays a spectral range similar to ICG and a 15–20-fold higher fluorescence signal. Additionally, the photostability after 1 h irradiation at 760 nm only has a minor decrease in fluorescence intensity. To date, NICE has been validated in multiple preclinical models [15,16,17,18,19].

Two techniques to coat medical devices (i.e., dip coating and paint brushing) have been published by our group [15]. In this study, we use the dip-coating method to coat 16 French catheters, which consists of three immersions of the catheter into the NICE with a period of drying between each immersion (coating time: roughly 1 h).

**Figure 2:**
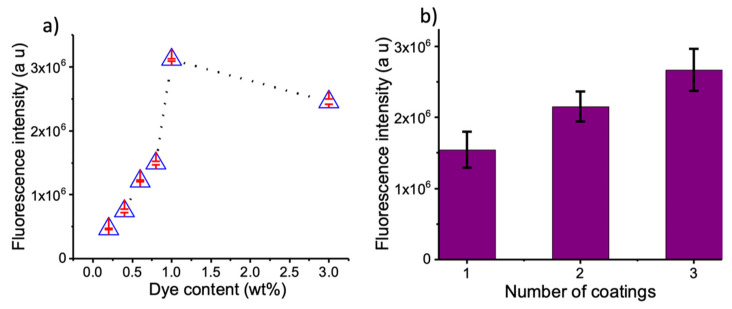
Near-infrared coating of equipment. (**a**) Emission response of the coatings with varying concentrations of dye. (**b**) Analysis of fluorescence intensity after each coating.

Fluorescence intensity of the coatings were measured at room temperature on an Edinburg FS5 spectrofluorometer. A polymer coating without dye was used as a baseline for all the measurements. NIR fluorescence images of the coatings were captured with the help of a home-made NIR imager setup equipped with a scientific grade sCMOS camera, 740 nm LED light source, and 835/70 nm band pass filter. Fluorescence images were analyzed and the fluorescence intensity was quantified with the help of ImageJ software (Figure 2).

### 2.3. Operating Setup and Experimental Workflow

#### Animal Model

A median laparotomy was performed. A portion of the small bowel (namely the jejunum) was then selected 200 cm away from the ligament of Treitz, followed by an enterotomy. After the introduction of the coated catheter, the entry hole was then closed with 3/0 polysorb in a running suture fashion. The lights of the operating room were turned off during imaging acquisition in order to reduce light interference. The image capture was performed through photos, first using the NIR-I imaging system, then followed by the NIR-II imaging system (acquisition time: roughly 60 s for each device). As for the NIR-I camera, a zero-degree laparoscope D-light TH102 camera H3-Z FI TC300 CCU (KARL STORZ, GmbH, Germany) was used as a control for the NIR-II camera KIS II (Kaer Labs, Nantes, France). The camera’s distal lens was placed at an approximately 20 cm distance to capture the entire surgical area of interest. A calibration aid, providing a constant fluorescence signal (Green Balance™ ICG Reference Card; Diagnostic Green; Aschheim–Dornach, Germany), was placed in the abdominal cavity, close the region of interest to provide a reference fluorescence value. This reference card was therefore used to compute the fluorescence intensity as arbitrary units (a.u.) to correct any potential distance bias.

The NIR-I camera has a standard white light observation that can be switched to an NIR-I mode using a footswitch pedal, which concurrently activates the NIR-I light source and a bypass filter. The NIR-II camera used was the NIR-II KIS II optical imaging system, which is designed for an open surgical setting. It works with a computer-based interface, which allows for the control of technical parameters, such as exposure time. Additionally, filters can be mounted onto the distal end of the lens, restricting the wavelength of the shutter. For this experiment, a 1200 nm filter was used with a preset 60 ms exposure time.

Similar to the image acquisition of the small bowel, a cold gastrotomy was performed at the posterior gastric wall. The gastric fluid was aspirated prior to catheter insertion, preventing image interference and catheter migration. As in the small bowel, the gastrotomy was closed using 3/0 polysorb. The pattern of image acquisition for NIR-I and NIR-II cameras was previously described.

**Figure 3:**
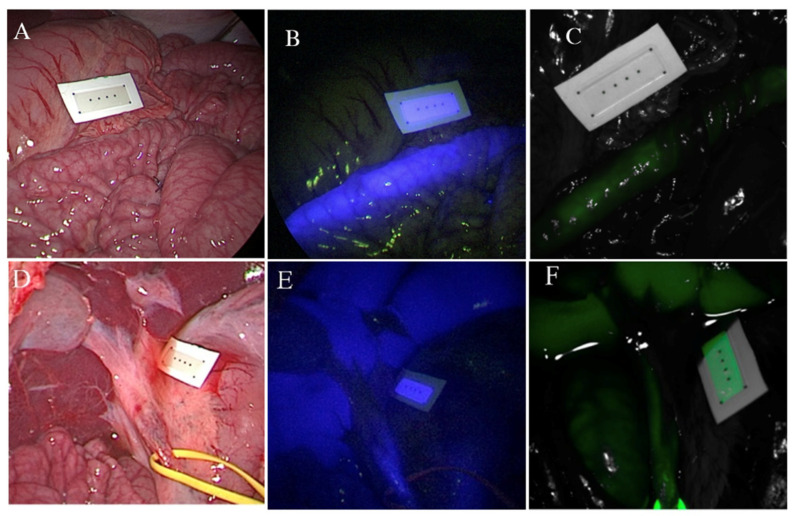
Visualization of the endoluminal small bowel NICE-coated catheter and CBD characterization at 35 min after ICG administration; (**A**) small bowel RGB image; (**B**) small bowel NIR-I window, overlay image (RGB + fluorescence); (**C**) small bowel NIR-II window, overlay (RGB + fluorescence) (**D**) CBD RGB image; (**E**) CBD NIR-I window, overlay image (RGB + fluorescence); (**F**) CBD NIR-II window, overlay (RGB + fluorescence). Abbreviations: RGB—red, green, blue; NIR—near-infrared; NICE—near-infrared coating of equipment, CBD—common bile duct, ICG—indocyanine green.

For the biliary procedure, the assistant surgeon exposed the CBD by retracting the gallbladder laterally. The surgeon then dissected the distal part of the CBD to ensure its proper skeletonization. After an adequate CBD dissection, the anesthesiologist intravenously infused the prepared ICG solution into a peripheral venous line. A 20 mL bolus of saline solution 0.9% was administered to ensure the ICG reached the systemic venous blood (Figure 3).

The first NIR image acquisition was achieved after 5 min (t5) of injection. The two NIR imaging systems were used as previously described. Further acquisitions were performed every 30 min for 1 h (t35 and t65). As for the endoluminal NICE-coated catheters, the external light interference was avoided during image acquisition. After t65, at the end of the procedure, biopsies were taken for histopathological analysis.

During all experiments, the ICG reference card was in the operative field next to the region of interest to compute the normalized fluorescence afterwards.

### 2.4. Post-Processing Fluorescence Analyses

Mean fluorescent intensities were calculated after the experimental analysis as total counts per ROI pixel. The OSIRIX Lite v12.0.1 imaging software (Pixmeo, Geneva, Switzerland) was used to quantify fluorescence intensity. We corrected the fluorescence by computing the relative fluorescence intensity as the ratio between the target and the adjacent reference card’s fluorescence intensity. As previously described, fluorescence intensity was defined in a.u. Areas with light scattering were avoided as ROI.

### 2.5. Statistical Analysis

Data were collected and recorded in a commercially available database (Excel spreadsheet in version 2021, Microsoft Corporation, Redmond, WA, USA) for subsequent statistical analysis. Data were reported as means and standard deviations (SD) unless otherwise indicated. Statistical comparisons between variables were made using a Student’s *t*-test for continuous or discrete variables. A *p*-value < 0.05 was considered statistically significant.

## 3. Results

The primary results are summarized in Table 1. In all animals, we were able to detect the MFI of endoluminal NICE-coated catheters and ICG during CBD characterization at different time points using the NIR-II imaging system.

All surgical procedures were performed without any adverse events.

## 4. Discussion

The ongoing research in optical imaging has been mostly dedicated to NIR-I (~700–900 nm). The present study analyzed the feasibility of NIR-I fluorescent dye bioimaging in the NIR-II window (1000–1700 nm) [10]. We were able quantify the fluorescence intensity of ICG and NICE at off-peak spectra, confirming their tail emission.

The NIR-II window has recently garnered attention since it is well-known that, at a longer wavelength after laser excitation, photons travel in the tissues with significantly less attenuation and scattering. The scattering of NIR-II photons (scaling with λ-α; α = 0.2–4 for most tissues) is therefore diminished, thereby providing a higher and more precise resolution [11]. To optimize FIGS within the NIR-II window, developments of cornerstone components such as fluorophores and optical imaging systems must be encouraged.

As for fluorophores, NIR-II nanomaterials have recently been developed (single-walled carbon nanotubes, quantum dots, and rare-earth-doped nanoparticles). For instance, Safranko et al. demonstrated high quantum yield and antioxidant activity in a sustainable biomass waste model that enabled ion sensing and cellular imaging of cancer cells [20].

However, the latest NIR-II organic dyes have mostly implemented a cyanine or electron donor-acceptor-donor (CH1055PEG ~1000–1700 nm) structure [21,22] in which the inorganic properties are of concern for clinical safety, since these materials can be accumulated within solid organs, such as the liver or spleen [10]. Consequently, NIR-II dyes are still in a development phase, and there are currently no NIR-II dyes which have been FDA-approved for clinical use.

As a result, there is a significant interest in evaluating validated NIR-I dyes proven to achieve a high quantum yield (QY) performance in the NIR-II window [11].

As previously described, ICG tail emission was unintentionality found in vitro [17]. It was later on studied by Starosolski et al. in an ICG perfusion study in mice models [12]. In the preclinical study, authors used a custom-built spectral NIR assembly to simultaneously assess imaging of the two NIR windows. Target-to-background ratios (TBRs) in the NIR-II imaging system almost double those of the NIR-I imaging system [12].

Siqi Gao et al. recently published a large animal study using a clinically available and improved NIR-Ι/II multispectral imaging system. Authors simultaneously assessed ICG at 800 nm and 1100 nm wavelengths to map the microvascular network and assess the perfusion status on a skin avulsion–injury model [23].

They used an upgraded commercially available imagining system that was used in the first in-human ICG-guided liver resection of primary and metastatic liver tumors in 23 patients in 2019. This study found that NIR-II imaging provided a higher tumor-detection sensitivity (100% vs. 90.6%), a higher tumor to normal liver tissue signal ratio (5.33 vs. 1.45) and an enhanced tumor detection rate (56.41% vs. 46.15%) [24].

These pivotal reports have provided a plausible foundation for our current experiment, which detects the ICG MFI at the tail emission (1200 nm) in a large animal model using a recent commercially available NIR-II imaging system. Interestingly, the previously published NIR-II off-peak emission of ICG has even been detectable up to 1575 nm [17].

By assessing ICG fluorescence intensity at the tail emission, we were able to characterize the CBD at 5, 35, and 65 min. Failure to identify the anatomy seems to be one of the main factors related to bile duct injuries (BDIs), i.e., clinical scenarios of chronic inflammation [4]. FIGS in the NIR-I window has been vastly studied, thereby helping surgeons prevent BDIs [25]. Despite the efforts made as well as the introduction of advanced technology in this domain, including artificial intelligence, the incidence of BDIs remains stable. Future research in thickened inflamed tissue aiming to enhance FIGS in the NIR-II window is a subject of real interest [26].

Fluorophores, which can adhere to surgical materials, are attractive options as they do not necessitate any systemic administration, hence permitting faster clinical translation. NICE was developed as a coating dye alternative to ICG. Our research group has previously obtained excellent results in terms of a constant spectral signal while experimenting in preclinical models. To date, NICE-coated medical devices have been used to intraoperatively identify the ureter and the urethra [19] for gastrointestinal clip marking (over-the-scope clips (OVESCO)) [14] and to guide magnetic anastomotic devices during endolaparoscopic gastrojejunostomies [18]. Our previous studies evaluating NICE have generally been performed within the NIR-I window. Since NICE is a cyanine 7.5 derivate and as the emission wavelength is comparable to ICG, it was appropriate for evaluating NICE performance in the NIR-II domain [16].

Comparable to CBD characterization, endoluminal NICE-coated catheters in the small bowel and the stomach were visualized in all animals using the NIR-II imaging system, hence illustrating NICE fluorescence intensity at the tail emission. In reality, NICE achieves a superior brightness (15–20-fold higher) compared to ICG [16]. The next sensible step in research would be to evaluate NICE within deeper anatomical tissues.

The NIR-II window is still in its infancy compared to the vast evolution found in NIR-I bioimaging. Contrary to NIR-I cameras, charge-coupled devices (CCD) cannot be used in NIR-II imaging systems since they are largely insensitive to wavelengths over 1000 nm [26]. Consequently, NIR-II systems require the use of more sophisticated and higher-cost detectors, such as a compound semiconductor build-up of InGaAs or HgCdTe [27]. At present, the majority of NIR-II imaging systems have been engineered for fluorescence microscopy, some of them customized for preclinical studies, and very few are available in clinical settings [11].

The merit of our study lies in the fact that this preclinical trial evaluates the fluorescence intensity at the tail emission of two NIR-I dyes. While characterizing anatomical structures of interest in all porcine models, ICG and NICE prove to have adequate performance in the NIR-II domain.

The limitation of our study is represented by the small sample size. In addition, although it was beyond the scope of the study’s objective, we did not find any superiority when comparing the two NIR imaging systems, which have been claimed by the previous NIR-II ex vivo and in vivo studies, as well as other clinical trials. We acknowledge that data have been collected from different anatomical sites, where differences in tissue structure could affect the yield of the emission signal in the two NIR-I and NIR-II windows; therefore the results should be considered with caution.

## 5. Conclusions

The in vivo assessment of ICG and NICE fluorescence intensity at the tail emission is feasible, hence allowing for CBD discrimination and identification of endoluminal NICE-coated surgical catheters in a large animal model. This study supports the previously reported accurate in vivo performance of ICG in the NIR-II window at longer wavelengths (1200 nm), and it represents NICE assessment in the NIR-II domain for the first time, providing a foundation for further preclinical confirmatory trials.

## Figures and Tables

**Table 1:** Fluorescent signal intensity expressed as arbitrary units (a.u.) identifying endoluminal NICE-coated catheters and CBD characterization after ICG administration.

	NIR-I Window	NIR-II Window	*p* Value
**Endoluminal NICE-coated catheters in the small bowel**	0.34 ± 0.007 a.u.	0.14 ± 0.106 a.u.	*p* = 0.111
**Endoluminal NICE-coated catheters in the stomach**	0.26 ± 0.156 a.u.	0.09 ± 0.039 a.u.	*p* = 0.248
**CBD after intravenous ICG injection**	**t5** 0.52 ± 0.198 a.u.	0.12 ± 0.027 a.u.	*p* = 0.101
	**t35** 0.54 ± 0.216 a.u	0.18 ± 0.100 a.u	*p* = 0.129
	**t65** 0.62 ± 0.251 a.u.	0.22 ± 0.041 a.u.	*p* = 0.151

Abbreviations: NIR—near-infrared, CBD—common bile duct, NICE—near-infrared coating of equipment.

## Data Availability

Data share upon reasonable request.

## References

[1] Hirche C., Engel H., Kolios L., Cognie J., Hünerbein M., Lehnhardt M., Kremer T. (2013). An experimental study to evaluate the fluobeam 800 imaging system for fluorescence-guided lymphatic imaging and sentinel node biopsy. Surg. Innov..

[2] Yamashita S., Tokuishi K., Anami K., Miyawaki M., Moroga T., Kamei M., Suehiro S., Ono K., Takeno S., Chujo M. (2011). Video-assisted thoracoscopic indocyanine green fluorescence imaging system shows sentinel lymph nodes in non-small-cell lung cancer. J. Thorac. Cardiovasc. Surg..

[3] Meershoek P., KleinJan G.H., van Willigen D.M., Bauwens K.P., Spa S.J., van Beurden F., van Gennep E.J., Mottrie A.M., van der Poel H.G., Buckle T. (2020). Multi-wavelength fluorescence imaging with a da Vinci Firefly—A technical look behind the scenes. J. Robot. Surg..

[4] Bsc L.V.M., Handgraaf H.J.M., Diana M., Dijkstra J., Ishizawa T., Vahrmeijer A.L., Mieog J.S.D. (2018). A practical guide for the use of indocyanine green and methylene blue in fluorescence-guided abdominal surgery. J. Surg. Oncol..

[5] Frangioni J.V. (2003). In vivo near-infrared fluorescence imaging. Curr. Opin. Chem. Biol..

[6] Yu Z., Eich C., Cruz L.J. (2020). Recent Advances in Rare-Earth-Doped Nanoparticles for NIR-II Imaging and Cancer Theranostics. Front. Chem..

[7] Vahrmeijer A.L., Hutteman M., Van Der Vorst J.R., Van De Velde C.J.H., Frangioni J.V. (2013). Image-guided cancer surgery using near-infrared fluorescence. Nat. Rev. Clin. Oncol..

[8] FDA (2021). Drugs @ FDA: FDA-Approved Drugs. In Prod. Inser. Indocyanine Green. https://www.accessdata.fda.gov/scripts/cder/daf/index.cfm?event=overview.process&applno=011525.

[9] Diana M., Agnus V., Halvax P., Liu Y.-Y., Dallemagne B., Schlagowski A.-I., Geny B., Diemunsch P., Lindner V., Marescaux J. (2015). Intraoperative fluorescence-based enhanced reality laparoscopic real-time imaging to assess bowel perfusion at the anastomotic site in an experimental model. Br. J. Surg..

[10] Spota A., Al-Taher M., Felli E., Conde S.M., Dal Dosso I., Moretto G., Spinoglio G., Baiocchi G., Vilallonga R., Impellizzeri H. (2021). Fluorescence-based bowel anastomosis perfusion evaluation: Results from the IHU-IRCAD-EAES EURO-FIGS registry. Surg. Endosc..

[11] Zhu S., Yung B.C., Chandra S., Niu G., Antaris A.L., Chen X. (2018). Near-Infrared-II (NIR-II) Bioimaging via Off-Peak NIR-I Fluorescence Emission. Theranostics.

[12] Starosolski Z., Bhavane R., Ghaghada K.B., Vasudevan S.A., Kaay A., Annapragada A. (2017). Indocyanine green fluorescence in second near-infrared (NIR-II) window. PLoS ONE.

[13] Kilkenny C., Browne W.J., Cuthill I.C., Emerson M., Altman D.G. (2010). Improving Bioscience Research Reporting: The ARRIVE Guidelines for Reporting Animal Research. PLoS Biol..

[14] Prescott M.J., Lidster K. (2017). Improving quality of science through better animal welfare: The NC3Rs strategy. Lab. Anim..

[15] Barberio M., Pizzicannella M., Spota A., Ashoka A.H., Agnus V., Al Taher M., Jansen-Winkeln B., Gockel I., Marescaux J., Swanström L. (2020). Preoperative endoscopic marking of the gastrointestinal tract using fluorescence imaging: Submucosal indocyanine green tattooing versus a novel fluorescent over-the-scope clip in a survival experimental study. Surg. Endosc..

[16] Ashoka A.H., Kong S.-H., Seeliger B., Andreiuk B., Soares R.V., Barberio M., Diana M., Klymchenko A.S. (2020). Near-infrared fluorescent coatings of medical devices for image-guided surgery. Biomaterials.

[17] Barberio M., Al-Taher M., Felli E., Ashoka A.H., Marescaux J., Klymchenko A., Diana M. (2021). Intraoperative ureter identification with a novel fluorescent catheter. Sci. Rep..

[18] Watanabe R., Barberio M., Kanaji S., Lapergola A., Ashoka A.H., Andreiuk B., Guerriero L., Pizzicannella M., Seeliger B., Saida Y. (2019). Hybrid fluorescent magnetic gastrojejunostomy: An experimental feasibility study in the porcine model and human cadaver. Surg. Endosc..

[19] Barberio M., Al-Taher M., Forgione A., Ashoka A.H., Felli E., Agnus V., Marescaux J., Klymchenko A., Diana M. (2020). A novel method for near-infrared fluorescence imaging of the urethra during perineal and transanal surgery: Demonstration in a cadaveric model. Color. Dis..

[20] Šafranko S., Stanković A., Hajra S., Kim H.-J., Strelec I., Dutour-Sikirić M., Weber I., Bosnar M.H., Grbčić P., Pavelić S.K. (2021). Preparation of Multifunctional N-Doped Carbon Quantum Dots from *Citrus clementina* Peel: Investigating Targeted Pharmacological Activities and the Potential Application for Fe^3+^ Sensing. Pharmaceuticals.

[21] Zhu S., Tian R., Antaris A.L., Chen X., Dai H. (2019). Near-Infrared-II Molecular Dyes for Cancer Imaging and Surgery. Adv. Mater..

[22] Buddingh K.T., Nieuwenhuijs V.B., van Buuren L., Hulscher J.B.F., de Jong J.S., van Dam G.M. (2011). Intraoperative assessment of biliary anatomy for prevention of bile duct injury: A review of current and future patient safety interventions. Surg. Endosc..

[23] Gao S., Yu Y., Wang Z., Wu Y., Qiu X., Jian C., Yu A. (2022). NIR-II Fluorescence Imaging Using Indocyanine Green Provides Early Prediction of Skin Avulsion-Injury in a Porcine Model. Clin. Cosmet. Investig. Dermatol..

[24] Hu Z., Fang C., Li B., Zhang Z., Cao C., Cai M., Su S., Sun X., Shi X., Li C. (2020). First-in-human liver-tumour surgery guided by multispectral fluorescence imaging in the visible and near-infrared-I/II windows. Nat. Biomed. Eng..

[25] Lin M., Chen L., Huang Z., Qiu H., Yu B. (2019). Neutrophils injure gallbladder interstitial Cajal-like cells in a guinea pig model of acute cholecystitis. J. Cell. Physiol..

[26] Zhu B., Sevick-Muraca E.M. (2015). A review of performance of near-infrared fluorescence imaging devices used in clinical studies. Br. J. Radiol..

[27] Oduor P., Mizuno G., Lewis J. (2016). Strategic options towards an affordable high-performance infrared camera. Image Sens. Technol. Mater. Devices Syst. Appl. III.

